# Open Targets Platform: facilitating therapeutic hypotheses building in drug discovery

**DOI:** 10.1093/nar/gkae1128

**Published:** 2024-12-06

**Authors:** Annalisa Buniello, Daniel Suveges, Carlos Cruz-Castillo, Manuel Bernal Llinares, Helena Cornu, Irene Lopez, Kirill Tsukanov, Juan María Roldán-Romero, Chintan Mehta, Luca Fumis, Graham McNeill, James D Hayhurst, Ricardo Esteban Martinez Osorio, Ehsan Barkhordari, Javier Ferrer, Miguel Carmona, Prashant Uniyal, Maria J Falaguera, Polina Rusina, Ines Smit, Jeremy Schwartzentruber, Tobi Alegbe, Vivien W Ho, Daniel Considine, Xiangyu Ge, Szymon Szyszkowski, Yakov Tsepilov, Maya Ghoussaini, Ian Dunham, David G Hulcoop, Ellen M McDonagh, David Ochoa

**Affiliations:** Open Targets, Wellcome Genome Campus, Hinxton, Cambridgeshire CB10 1SD, UK; European Molecular Biology Laboratory, European Bioinformatics Institute (EMBL-EBI), Wellcome Genome Campus, Hinxton, Cambridgeshire CB10 1SD, UK; Open Targets, Wellcome Genome Campus, Hinxton, Cambridgeshire CB10 1SD, UK; European Molecular Biology Laboratory, European Bioinformatics Institute (EMBL-EBI), Wellcome Genome Campus, Hinxton, Cambridgeshire CB10 1SD, UK; Open Targets, Wellcome Genome Campus, Hinxton, Cambridgeshire CB10 1SD, UK; European Molecular Biology Laboratory, European Bioinformatics Institute (EMBL-EBI), Wellcome Genome Campus, Hinxton, Cambridgeshire CB10 1SD, UK; Open Targets, Wellcome Genome Campus, Hinxton, Cambridgeshire CB10 1SD, UK; European Molecular Biology Laboratory, European Bioinformatics Institute (EMBL-EBI), Wellcome Genome Campus, Hinxton, Cambridgeshire CB10 1SD, UK; Open Targets, Wellcome Genome Campus, Hinxton, Cambridgeshire CB10 1SD, UK; European Molecular Biology Laboratory, European Bioinformatics Institute (EMBL-EBI), Wellcome Genome Campus, Hinxton, Cambridgeshire CB10 1SD, UK; Open Targets, Wellcome Genome Campus, Hinxton, Cambridgeshire CB10 1SD, UK; European Molecular Biology Laboratory, European Bioinformatics Institute (EMBL-EBI), Wellcome Genome Campus, Hinxton, Cambridgeshire CB10 1SD, UK; Open Targets, Wellcome Genome Campus, Hinxton, Cambridgeshire CB10 1SD, UK; European Molecular Biology Laboratory, European Bioinformatics Institute (EMBL-EBI), Wellcome Genome Campus, Hinxton, Cambridgeshire CB10 1SD, UK; Open Targets, Wellcome Genome Campus, Hinxton, Cambridgeshire CB10 1SD, UK; European Molecular Biology Laboratory, European Bioinformatics Institute (EMBL-EBI), Wellcome Genome Campus, Hinxton, Cambridgeshire CB10 1SD, UK; Open Targets, Wellcome Genome Campus, Hinxton, Cambridgeshire CB10 1SD, UK; European Molecular Biology Laboratory, European Bioinformatics Institute (EMBL-EBI), Wellcome Genome Campus, Hinxton, Cambridgeshire CB10 1SD, UK; Open Targets, Wellcome Genome Campus, Hinxton, Cambridgeshire CB10 1SD, UK; European Molecular Biology Laboratory, European Bioinformatics Institute (EMBL-EBI), Wellcome Genome Campus, Hinxton, Cambridgeshire CB10 1SD, UK; Open Targets, Wellcome Genome Campus, Hinxton, Cambridgeshire CB10 1SD, UK; European Molecular Biology Laboratory, European Bioinformatics Institute (EMBL-EBI), Wellcome Genome Campus, Hinxton, Cambridgeshire CB10 1SD, UK; Open Targets, Wellcome Genome Campus, Hinxton, Cambridgeshire CB10 1SD, UK; European Molecular Biology Laboratory, European Bioinformatics Institute (EMBL-EBI), Wellcome Genome Campus, Hinxton, Cambridgeshire CB10 1SD, UK; Open Targets, Wellcome Genome Campus, Hinxton, Cambridgeshire CB10 1SD, UK; European Molecular Biology Laboratory, European Bioinformatics Institute (EMBL-EBI), Wellcome Genome Campus, Hinxton, Cambridgeshire CB10 1SD, UK; Open Targets, Wellcome Genome Campus, Hinxton, Cambridgeshire CB10 1SD, UK; European Molecular Biology Laboratory, European Bioinformatics Institute (EMBL-EBI), Wellcome Genome Campus, Hinxton, Cambridgeshire CB10 1SD, UK; Open Targets, Wellcome Genome Campus, Hinxton, Cambridgeshire CB10 1SD, UK; European Molecular Biology Laboratory, European Bioinformatics Institute (EMBL-EBI), Wellcome Genome Campus, Hinxton, Cambridgeshire CB10 1SD, UK; AstraZeneca UK Limited; Open Targets, Wellcome Genome Campus, Hinxton, Cambridgeshire CB10 1SD, UK; European Molecular Biology Laboratory, European Bioinformatics Institute (EMBL-EBI), Wellcome Genome Campus, Hinxton, Cambridgeshire CB10 1SD, UK; Open Targets, Wellcome Genome Campus, Hinxton, Cambridgeshire CB10 1SD, UK; European Molecular Biology Laboratory, European Bioinformatics Institute (EMBL-EBI), Wellcome Genome Campus, Hinxton, Cambridgeshire CB10 1SD, UK; Open Targets, Wellcome Genome Campus, Hinxton, Cambridgeshire CB10 1SD, UK; European Molecular Biology Laboratory, European Bioinformatics Institute (EMBL-EBI), Wellcome Genome Campus, Hinxton, Cambridgeshire CB10 1SD, UK; Open Targets, Wellcome Genome Campus, Hinxton, Cambridgeshire CB10 1SD, UK; European Molecular Biology Laboratory, European Bioinformatics Institute (EMBL-EBI), Wellcome Genome Campus, Hinxton, Cambridgeshire CB10 1SD, UK; Open Targets, Wellcome Genome Campus, Hinxton, Cambridgeshire CB10 1SD, UK; Wellcome Sanger Institute, Wellcome Genome Campus, Hinxton, Cambridgeshire CB10 1SA, UK; Open Targets, Wellcome Genome Campus, Hinxton, Cambridgeshire CB10 1SD, UK; European Molecular Biology Laboratory, European Bioinformatics Institute (EMBL-EBI), Wellcome Genome Campus, Hinxton, Cambridgeshire CB10 1SD, UK; Open Targets, Wellcome Genome Campus, Hinxton, Cambridgeshire CB10 1SD, UK; European Molecular Biology Laboratory, European Bioinformatics Institute (EMBL-EBI), Wellcome Genome Campus, Hinxton, Cambridgeshire CB10 1SD, UK; Open Targets, Wellcome Genome Campus, Hinxton, Cambridgeshire CB10 1SD, UK; Wellcome Sanger Institute, Wellcome Genome Campus, Hinxton, Cambridgeshire CB10 1SA, UK; Open Targets, Wellcome Genome Campus, Hinxton, Cambridgeshire CB10 1SD, UK; Wellcome Sanger Institute, Wellcome Genome Campus, Hinxton, Cambridgeshire CB10 1SA, UK; Open Targets, Wellcome Genome Campus, Hinxton, Cambridgeshire CB10 1SD, UK; Wellcome Sanger Institute, Wellcome Genome Campus, Hinxton, Cambridgeshire CB10 1SA, UK; Open Targets, Wellcome Genome Campus, Hinxton, Cambridgeshire CB10 1SD, UK; Wellcome Sanger Institute, Wellcome Genome Campus, Hinxton, Cambridgeshire CB10 1SA, UK; Open Targets, Wellcome Genome Campus, Hinxton, Cambridgeshire CB10 1SD, UK; Wellcome Sanger Institute, Wellcome Genome Campus, Hinxton, Cambridgeshire CB10 1SA, UK; Open Targets, Wellcome Genome Campus, Hinxton, Cambridgeshire CB10 1SD, UK; European Molecular Biology Laboratory, European Bioinformatics Institute (EMBL-EBI), Wellcome Genome Campus, Hinxton, Cambridgeshire CB10 1SD, UK; Wellcome Sanger Institute, Wellcome Genome Campus, Hinxton, Cambridgeshire CB10 1SA, UK; Open Targets, Wellcome Genome Campus, Hinxton, Cambridgeshire CB10 1SD, UK; European Molecular Biology Laboratory, European Bioinformatics Institute (EMBL-EBI), Wellcome Genome Campus, Hinxton, Cambridgeshire CB10 1SD, UK; Wellcome Sanger Institute, Wellcome Genome Campus, Hinxton, Cambridgeshire CB10 1SA, UK; Open Targets, Wellcome Genome Campus, Hinxton, Cambridgeshire CB10 1SD, UK; European Molecular Biology Laboratory, European Bioinformatics Institute (EMBL-EBI), Wellcome Genome Campus, Hinxton, Cambridgeshire CB10 1SD, UK; Wellcome Sanger Institute, Wellcome Genome Campus, Hinxton, Cambridgeshire CB10 1SA, UK; Open Targets, Wellcome Genome Campus, Hinxton, Cambridgeshire CB10 1SD, UK; European Molecular Biology Laboratory, European Bioinformatics Institute (EMBL-EBI), Wellcome Genome Campus, Hinxton, Cambridgeshire CB10 1SD, UK

## Abstract

The Open Targets Platform (https://platform.opentargets.org) is a unique, open-source, publicly-available knowledge base providing data and tooling for systematic drug target identification, annotation, and prioritisation. Since our last report, we have expanded the scope of the Platform through a number of significant enhancements and data updates, with the aim to enable our users to formulate more flexible and impactful therapeutic hypotheses. In this context, we have completely revamped our target–disease associations page with more interactive facets and built-in functionalities to empower users with additional control over their experience using the Platform, and added a new Target Prioritisation view. This enables users to prioritise targets based upon clinical precedence, tractability, doability and safety attributes. We have also implemented a direction of effect assessment for eight sources of target–disease association evidence, showing the effect of genetic variation on the function of a target is associated with risk or protection for a trait to inform on potential mechanisms of modulation suitable for disease treatment. These enhancements and the introduction of new back and front-end technologies to support them have increased the impact and usability of our resource within the drug discovery community.

## Graphical Abstract

**Graphical Abstract:**
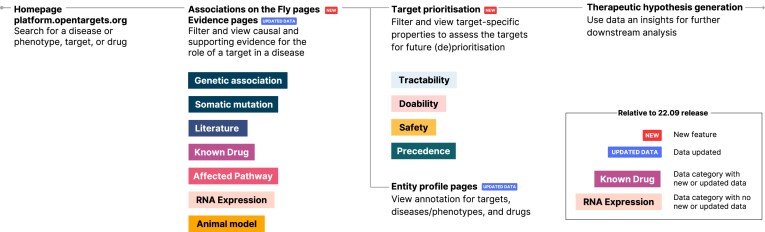

## Introduction

Despite many advances in drug discovery and development, our collective ability to develop safe and effective medicines is still limited by an incomplete understanding of disease biology. For every 10 drugs that enter clinical trials, only one will reach regulatory approval (1), with 79% of the programs closing due to efficacy or safety concerns (2). Studies indicate that an enhanced understanding of the disease-causal mechanisms at the target discovery stage can double the success rate and halve the early stoppage of clinical studies (3,4). However, the information required to formulate precise therapeutic hypotheses is still sparse, or we lack the understanding to convert the data into the knowledge necessary to inform decisions (5). In recent times, several resources have attempted to consolidate the publicly available information into web services aimed to assist the identification of drug targets using a diverse set of sources for one or several therapeutic areas (6–9).

Open Targets (https://www.opentargets.org/) is a pre-competitive partnership combining expertise from academia and the pharmaceutical industry to support the systematic identification and prioritisation of drug targets. One axis of the consortium's research programme provides open-source data and informatics tools for the global scientific community, with the Open Targets Platform (https://platform.opentargets.org/) as its flagship resource (10). In our last NAR update, we described how the Platform was undergoing a complete rebuild, aiming to streamline data integration and harmonisation, expand users’ data exploration, and improve the user experience (10). This enabled us to develop features for users to more dynamically prioritise targets and build therapeutic hypotheses beyond the evidence for target–disease association to progressability factors. Here, we describe the significant changes and improvements we have implemented in the Open Targets Platform since our last database update, covering data updates, frontend tools, backend technologies and usability enhancements.

## Enhancing the ability to identify, explore and prioritise target–disease associations

**Figure 1.**
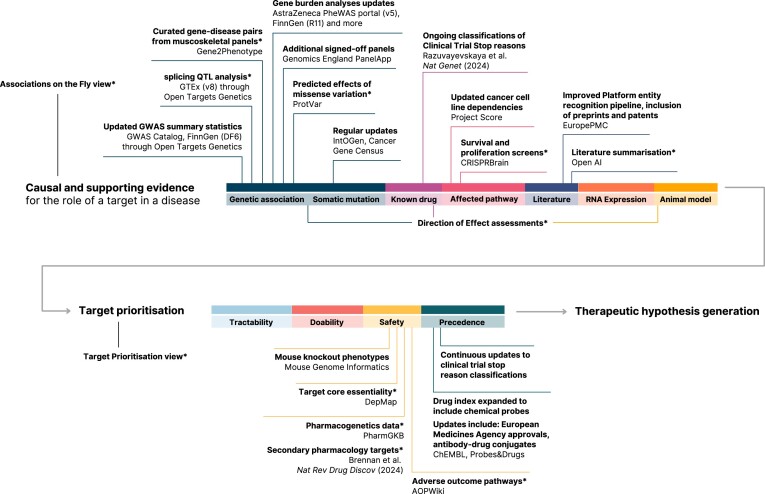
The Open Targets Platform integrates data informing multiple steps in the target identification and prioritisation process, from assessing the causal and supporting evidence of a target's role in disease through target prioritisation to therapeutic hypothesis generation. Here, we show where we have updated and integrated data along this journey. * indicates new data or features.

Establishing a causal link between the drug target and the indication constitutes a fundamental step in drug discovery. The Open Targets Platform aims to contextualise the available evidence by describing the relationship between targets and diseases carefully curated in 23 independent public sources (11). These sources cover different angles of our understanding of disease biology, spanning germline or somatic variation, perturbation experiments in cellular or animal models, affected pathways, known drugs or clinical candidates, and information extracted from literature mining. Targets and diseases are mapped to Ensembl gene identifiers (12) and Experimental Factor Ontology terms (13), respectively, and evidence is scored and aggregated to build a ranked list of associations summarising our confidence in the causal relationship between both entities (11,14). In Figure 1, we provide an overview of new sources of evidence and annotation integrated into the Platform since our last update, which contribute to this causal and supporting evidence for target–disease associations. Complementary to this, Supplementary Table S1 provides a more quantitative view of the Platform data expansion since the last report.

### Enhancing common and rare variation evidence for target–disease associations

Distinctive genetic variations associated with a given disease or phenotype can be leveraged to establish a likely causal mechanism leading to the identification of novel drug targets (15). Retrospective analysis indicates that genetic evidence can be found for up to two-thirds of FDA approvals (16). Thus, the Platform aims to systematically integrate publicly available human germline and somatic genetic variation associated with diseases or traits, centralising evidence of varying allelic frequencies reported in different resources for target prioritisation.

Since the last update, we have expanded our understanding of common disease genetics by including the latest Open Targets Genetics post-genome-wide association studies (GWAS) analysis data release (17). This iteration analyses 6591 additional GWAS Catalog studies from 70 publications, as well as summary statistics and credible sets from 2861 endpoints phenotyped across 260 000 individuals in the FinnGen data freeze 6 (18). Moreover, we expanded the GWAS/molecular trait colocalisation results by including splicing QTLs from GTEx version 8 (19), increasing our power to nominate likely causal genes through the Locus2Gene (L2G) machine learning method (20).

Interpreting rare protein-coding events through gene-level analysis complements the ability of GWAS to depict the genetic architecture of common conditions. Recently, we expanded our curation of Gene Burden results to include a selection of large scale cohort studies. For example, we updated the gene-level analysis from AstraZeneca's PheWAS Portal to version 5, enhancing the collapsing analysis derived from 470 000 individuals in the UKBiobank (21). Other updates to our Gene Burden evidence include evidence informing about circulating metabolic biomarkers for cardiovascular disease from the INTERVAL cohort (22), schizophrenia from the SCHEMA consortium (23), Parkinson's disease from AMP-PD (24), ancestry-specific evidence for prostate cancer in Black South African men (25) and the new FinnGen R11 dataset (18). In total, our Gene Burden data updates resulted in 2584 new gene-disease associations not previously covered by our gene burden or GWAS results.

By definition, rare diseases occur in fewer than 1 in 2000 individuals and are often driven by rare genetic variants; therefore, dedicated resources aim to capture the genes involved in clinically characterised diseases, preserving the privacy of individual genotypes. Among other updates since the last NAR report, the Platform now includes 173 signed-off panels related to genomic tests listed in the NHS National Genomic Test Directory, as reported by Genomics England PanelApp (26). Updates from Gene2Phenotype now include clinical curation of musculoskeletal panels, bringing the list of curated gene-disease pairs to 2697 (27). Further improvements to the ClinVar ingestion pipeline throughout this period resulted in the swift integration of germline and somatic variation submissions fully mapped to EFO (28,29). Additional disease-causing somatic variation was expanded to cover additional curation by the Cancer Gene Census and cancer driver gene predictions derived from 48 new cohorts performed by IntOGen (30,31).

### Understanding disease-associated genes using cellular perturbation screenings

Experimental perturbation of genes in cellular models through techniques like CRISPR can provide a complementary readout to the effect of natural variation from population genetics. Perturbation of a gene/protein can inform on its function and its role in disease mechanism, providing evidence for whether and how to potentially modulate it for the treatment of a disease. Since the last update, we have incorporated the second generation of Project Score, a map of cancer dependencies derived from the combination of multi-omic data, molecular markers and dependencies observed in 771 cancer cell lines (32). This study expands upon the original observation that synthetic-lethality can be used to nominate cancer dependencies, and a key finding from the Project Score project revealed WRN helicase as a potential target in microsatellite unstable tumours (11,32). Additionally, we also included 23 survival and proliferation screens from CRISPRbrain (33), an open-access platform harmonising functional genomics screens in iPSC differentiated into neuronal cells. The phenotypic readouts from these models constitute relevant evidence for 7 unique diseases in the ontology. 33 579 (73.7%) of the 45 507 indirect gene-disease associations derived from CRISPRbrain represent new associations not reported by any germline data sources, and 15 243 of them (33.5%) are entirely novel associations, not supported by any other data source, demonstrating the potential value of orthogonal information informing the effects of gene modulation.

### Introducing a better understanding of the effect of modulation and direction of disease-causing mechanisms

**Figure 2.**
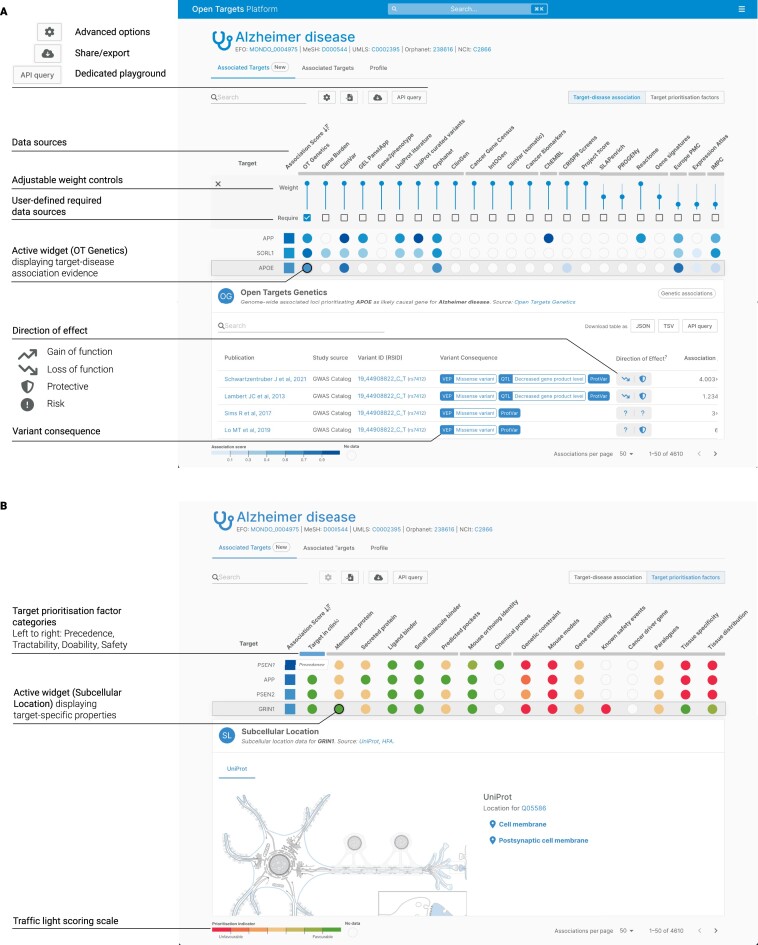
Summary panel of Associations on the Fly user interface and Target Prioritisation View. (**A**) Example Associations on the Fly view for Alzheimer's Disease [https://platform.opentargets.org/disease/MONDO_0004975/associations], showing all targets associated with the disease, sorted by decreasing association score (from darker to lighter blue). All the main features are labelled, including direction of effect and variant consequence information that provide a more mechanistic context to target–disease associations. (**B**) Example target prioritisation view ‘traffic light’ system for target prioritisation factors [https://platform.opentargets.org/disease/MONDO_0004975/associations?table=prioritisations], with darker green as the most favourable moving through to deeper red as the least favourable (with annotation bars). As highlighted in the text, a target located in the cell membrane or plasma membrane will be green in this view as it is likely to be more accessible to a drug and thus favourable (GRIN1 example).

Understanding the extent and direction in which a gene or protein modulation affects the disease or phenotype is essential in formulating therapeutic hypotheses. For example, where a reduction in protein activity confers protection for a disease, an inhibitor drug may benefit the patient population. Conversely, where an increase in protein activity confers protection for a disease, an agonist might represent a more suitable hypothesis. Since the last update, we have assessed all of our current sources that might inform the direction-of-effect to assist in this decision. In every evidence widget with available assessments, we now present an indicative suggestion on the likely directionality of the effect on the gene/protein expression/function; gain-of-function, loss-of-function, or inconclusive, as well as of the likely impact on the trait due to this effect; protect, risk or inconclusive (Figure 2A). A total of 2.3 million assessments corresponding to 865, 816 unique target–disease pairs were performed across eight different data sources to interpret the nature of the evidence in terms of its impact on gene/protein function (gain-of-function/Loss-of-function) and disease impact (protective/risk). For example, all evidence derived from IMPC mouse knockouts (34) was considered loss-of-function-risk, while an inhibitor drug that has passed through clinical evaluation would be regarded as loss-of-function-protective. A full description of the rules used to perform each assessment is available in the Platform documentation [https://platform-docs.opentargets.org/evidence].

To better understand the effect of coding variation on protein function, we have also incorporated a link to EMBL-EBI ProtVar in all evidence widgets, which contain genetic variants. This new resource, funded through an Open Targets project (35) (Figure 2A), includes predicted effects of missense variation based on AlphaMissense (36), interaction interfaces derived from AlphaFold (37) and protein abundance and stability based on protein structures using FoldX (38). This annotation complements the Ensembl VEP annotations previously displayed on all our variant-based evidence (27). A better understanding of the variant effect on genes and proteins and its directional effect on gene function and trait expands our knowledge of the target–disease relationship, offering new avenues to think about therapeutic intervention.

### Extracting biomedical knowledge from the scientific literature

A large fraction of the knowledge that can help nominate drug targets for particular indications remains in unstructured text. The Platform has continued to expand its capacity to extract information from text resources by starting to mine preprints and patents included in the EuropePMC corpus (39). Enhancements to our natural entity recognition and normalisation pipelines have resulted in extraction of 440 million recognised entities from over 14.7 million publications (Tirunagari S, Saha S, Venkatesan A, Suveges D, Buniello A, Ochoa D, *et al.* Lit-OTAR Framework for Extracting Biological Evidences from Literature. bioRxiv. 2024. p. 2024.03.06.583722). Additionally, we continue to report and score 73.6 million target–disease co-occurrences, building additional evidence to support target nomination. To enhance how users digest this vast amount of information, we have developed new features within the Bibliography widget, such as filtering the mined literature by date.

Further, the Platform now leverages OpenAI’s ‘GPT4o-mini’ model to summarise target–disease evidence from all data sources when a full-text article is available (40). In detail, the Platform creates a chain of queries to the Open AI API using Langchain, providing the publication content as context. The relationship between a target and a disease is summarised with the following prompt: ‘Can you provide a concise summary about the relationship between [target] and [disease] according to this study?’. The resulting text is presented to the user. For example, when a user browses through literature evidence for association between *CFTR* and cystic fibrosis, this new feature can summarise the gene-disease relationship, providing context beyond the scope of an abstract by utilising the full-text article when available (see also our dedicated documentation page). This feature is also accessible through the API. When we initially developed the tool we chose to adopt GPT-3.5 due to the easy integration of the OpenAI API, which also provided accurate results based on empirical quality control at a relatively low cost. As part of our benchmarking effort, we have migrated to GPT-4o-mini mainly due to better performance from the larger LLM context window.

### Associations on the Fly

Designed and built upon extensive user experience, the new ‘Associations on the Fly’ dashboard allows the Platform user to customise our disease and target association views to help answer more advanced therapeutic hypotheses queries (Figure 2). In the recently introduced data source-based heatmap, the user can alter the relative weight of individual evidence sources (11) based on their relevance to the specific user question (evidence with expected weaker causal links are down-weighted by default). This results in dynamically generated association scores that update the ranked lists. For example, users can require evidence from particular data sources, such as OT Genetics (Figure 2A). They can also exclude known drugs or clinical candidates in ChEMBL (41) to rank associations that ignore previous drug development efforts (Figure 2A). Moreover, the user can now click on the computed associations to immediately view and understand the underlying evidence from the primary source, such as the sample size of a GWAS Catalog study (42) (Figure 2A). As additional customisations, users can pin targets/diseases from the ranked associations to move them to the top of the list for review. Then, to allow users to start their journey with a predefined list of targets or diseases of interest, the associations page allows the upload of a list of entities in multiple compatible identifiers, including Ensembl, Uniprot, HGNC or Experimental Factor Ontology (7,8,43,42) (Figure 2A). Moreover, enhanced data-sharing capabilities through URL, text-file download, and API playground support the redesigned associations dashboard (Figure 2A).

## Progressing therapeutic hypothesis through target prioritisation

While many targets can present compelling evidence of association with a given disease, not all are equally amenable to therapeutic intervention. Potential drug targets initially identified through causal evidence or analogous hypothesis building require additional prioritisation for drug discovery based on their suitability for drug discovery pipelines. Evaluating the unfavourable and favourable factors for drug discovery is critical to understanding the risk-benefit of a given therapeutic strategy.

### Target prioritisation

The Platform recently introduced a Target Prioritisation view to complement the target–disease association view. Using a traffic light colouring schema, this heatmap captures pre-computed target-specific properties that could favour or disfavour drug development (Figure 2B). The target properties are classified based on whether the target has been drugged before (clinical precedence), information that might influence the selection of a specific modality (tractability), whether there are models, tools and/or reagents that allow target assessment in preclinical settings to enable exploration of a given target (doability), and whether there are likely risks to modulating a target (safety) (5). Each factor in each category is evaluated individually. For example, a target could be considered unfavourable if highly constrained in human populations according to gnomAD (44), if it presents dependencies across all CancerDepMap cell lines (32) or if it has poor tissue expression selectivity according to Expression Atlas and Human Proteome Atlas (45,46). Conversely, a molecule targeted with previously approved drugs for any indication, presenting high-quality predicted pockets or selectively expressed in the cell membrane (Figure 2B), will be regarded as a favourable choice as a drug target (41,43,46). While none of these factors would discard a therapeutic hypothesis alone, understanding the risks derived from otherwise disparate information helps to contextualise drug discovery decision-making.

### Hypothesis-building using clinical candidates and approved drugs

Selecting new strategies to discover safe and effective drug targets requires a comprehensive understanding of prior clinical efforts. Since the last update, the Platform has promptly reflected every drug or clinical candidate reported by all releases of the ChEMBL database, including updates in the mechanisms of action, black-box warnings or withdrawn status (47). All chemical probes in ChEMBL—as reported in Probes & Drugs Portal—qualify for inclusion in the Platform, raising the availability of potent and selective tool molecules for assay consideration (48). The curation of antibody-drug conjugates as a distinct modality has also enhanced the cellular context of the drug modulation. Additionally, the clinical indications sourced from ChEMBL now incorporate drug approvals from the European Medicines Agency, reporting 49 drugs exclusively authorised in Europe. Further enhancements of the clinical study information capture Early Phase I trials as a distinct category complementing ongoing efforts such as classifying clinical trial stop reasons retrieved from ClinicalTrials.gov. Overall, drug and clinical candidate annotation, their indications and side effects represent the closest information to the therapeutic context, potentially informing about all stages of our target prioritisation framework: precedence, tractability, doability, and safety.

### Drug responses and toxicity using pharmacogenetics

Since the last update, we have also introduced pharmacogenetic (PGx) evidence from PharmGKB, capturing the consequences of genetic variation on drug efficacy, dosing and adverse events (49). PharmGKB manually curates the evidence for a given variant and drug response, providing annotation for each patient genotype and an overarching level of evidence based on published literature and clinical guidelines. The Platform further enriches the PGx data by including Ensembl variant effect predictions, extracting drug response from the phenotype description, drug information, and whether the variation occurs in the direct drug target (12,27). The PGx data widget appears on drug and target pages to inform patients about stratification, responses, and toxicity. They also contribute to our target prioritisation view by informing the safety attributes (Figure 2B).

### Multi-modal target safety assessment

Nominating drug targets with lower risks of target safety can significantly impact portfolio management. The Platform collates several data sources informing about the possible safety consequences of target modulation. For example, we kept expanding the list of targets with well-characterised safety events. Well-characterised safety events are presented in the Safety widget, to which we added curated (experimentally validated) evidence from Adverse Outcome Pathways (Society for Advancement of AOPs, AOP-Wiki 2023). Available from aopwiki.org, a relevant publication including a list of commonly screened targets for secondary pharmacology (50), as well as genes in which variation causes increased toxicity according to PGx evidence. While the strength of evidence of the various data sources incorporated into the Safety widget varies, the 941 targets currently covered by the Safety widget compromise a set of targets with known safety events, making them unfavourable for pipeline progression. This dataset is available for download at https://platform.opentargets.org/downloads through our ‘target’ object.

In addition to the known safety events, the Target Prioritisation view presents a range of *in vitro* and *in vivo* features for ranking target safety. To better understand cellular gene essentiality, we have incorporated fitness dependencies exhibited when performing CRISPR-Cas9 genome-wide knock-out screenings on 900 cell lines, as reported by the Cancer DepMap (32). Targets catalogued as ‘Core Essential’ due to their pan-cancer dependencies are flagged as unfavourable targets in the Target Prioritisation view (Figure 2B). To complement this information with *in vivo* assays, we developed a score for the severity of observed phenotypes in mouse knock-outs (51). In addition, target RNA expression is ranked based on tissue distribution and specificity, further enabling targets to be flagged as unfavourable for safety.

## Towards a more FAIR platform

Built upon FAIR principles (52), the Open Targets Platform continues to adhere to its foundational open-source practices. Since the last update, we have updated the licence to CC0 v1.0, allowing unlimited access to our data (53). The Platform has now been included as a Microsoft Azure Open Dataset, further expanding Cloud accessibility for our data. The data pipelines and services have been consolidated, and technologies have been updated to minimise adoption restrictions, such as our recent migration to OpenSearch. Moreover, we have simplified the method for creating a standalone deployment, providing more accessible protocols for users generating their own version of the Open Targets Platform. To maximise contributions from the open-source community, standards have been raised across all Github repositories, allowing the development of third-party applications such as wrappers around our GraphQL APIs (54). Feedback on the infrastructure and business logic has been incorporated into data, backend, and frontend applications, the latter benefiting from extensive UX sessions. One example of a UX-driven feature is the redesigned Platform search, which now features entity descriptions, can be accessed through a keyboard shortcut, and provides a history of recent searches to assist users.

## Engaging the Open Targets community

The Open Targets Platform is dedicated to deepening our collective understanding of target identification and prioritisation by leveraging the expertise of our partner institutions and the increasingly collaborative community of users. Always supported by our extensive documentation (https://platform-docs.opentargets.org), we have continued to promote user engagement through outreach activities, up-to-date training materials, and the development of fora to collect user feedback. The Open Targets Community (https://community.opentargets.org) has grown in users and functionalities and now includes a dedicated feature requests section. For each release, new Platform features and data have been described on our blog (https://blog.opentargets.org/) and complemented with regular deep dives on significant topics, including gene burden analyses, pharmacogenetics, provenance metadata, and standalone deployment, as well as case studies to showcase how Platform data and code can be built on. Lastly, we have expanded our use of video tutorials to guide users through new functionalities, trying to maximise the accessibility and training experience for users with different backgrounds.

## Discussion and future plans

As the wealth of data grows in different domains to enhance our understanding of disease biology, there continues to be a need to integrate and evaluate the evidence that can identify promising drug targets and prioritise therapeutic hypotheses that will lead to new safe and effective medicines. Throughout the last two years, the Open Targets Platform has continued to progress its capabilities to provide a more complete and user-friendly view of the evidence that associates target to disease and the evidence that can be used for target prioritisation. New experimental data – such as the updated Project Score (55) or AstraZeneca's gene burden tests on 470k individuals (21) – have been rapidly integrated and presented for the immediate benefit of the global community. As new data modalities emerge, such as single-cell transcriptomics, the Platform has served as a gold standard for understanding the impact of additional layers of information on drug discovery and development (Dann E, Teeple E, Elmentaite R, Meyer KB, Gaglia G, Nestle F, *et al.* Single-cell RNA sequencing of human tissue supports successful drug targets. medRxiv. 2024. p. 2024.04.04.24305313). The overall harmonised data continues to be a public reference, enabling multiple retrospective studies on the value of human genetics for drug discovery (3,15,16,56). Several examples have also demonstrated the value of the Platform data in building more specialised applications or AI solutions (57,58). Ultimately, publicly available, structured datasets such as those offered by the Platform, can catalyse the development of further AI applications for early discovery (59).

The Platform's ability to rapidly assist hypotheses through the dynamic web interface enables a broad community of users to work on both broad and very specific questions. The Platform is often referenced as a gold-standard resource to help pinpoint previously identified genetic associations, known drugs, or clinical candidates and understand novel findings (60,61). The ability to address systematic queries and simultaneously enable more detailed, bespoke queries has been the focus of new feature development, resulting in the Associations on the Fly and Target Prioritisation views. Open Targets intention is to continue the development of these recently introduced frameworks, allow more tailored and context-specific queries, to expand on the availability of causal evidence and develop further refinements to showcase the factors that influence the prioritisation of drug targets for progression. One of the most significant upcoming efforts in this regard will be the tighter integration of Open Targets Genetics (17) to provide a more integrated, comprehensive view of common disease genetics within Open Targets Platform.

## Supplementary Material

gkae1128_Supplemental_File

## Data Availability

All data is publicly available for download here: [https://platform.opentargets.org/downloads] and from the EMBL-EBI FTP: [https://ftp.ebi.ac.uk/pub/databases/opentargets/platform/]. All code is available in GitHub (https://github.com/opentargets) and Zenodo (https://doi.org/10.5281/zenodo.14002231).

## Notes

Present address: Ellen M. McDonagh and David Ochoa, Open Targets and European Molecular Biology Laboratory – European Bioinformatics Institute (EMBL-EBI), Wellcome Genome Campus, Hinxton, Cambridgeshire CB10 1SD, UK.

## References

[1] Sun D. , Gao W., Hu H., Zhou S. Why 90% of clinical drug development fails and how to improve it?. Acta Pharm Sin B. 2022; 12:3049–3062. 35865092 10.1016/j.apsb.2022.02.002 PMC9293739

[2] Dowden H. , Munro J. Trends in clinical success rates and therapeutic focus. Nat. Rev. Drug Discov. 2019; 18:495–496. 31267067 10.1038/d41573-019-00074-z

[3] Minikel E.V. , Painter J.L., Dong C.C., Nelson M.R. Refining the impact of genetic evidence on clinical success. Nature. 2024; 629:624–629. 38632401 10.1038/s41586-024-07316-0 PMC11096124

[4] Razuvayevskaya O. , Lopez I., Dunham I., Ochoa D. Genetic factors associated with reasons for clinical trial stoppage. Nat. Genet. 2024; 56:1862–1867. 39075208 10.1038/s41588-024-01854-z PMC11387188

[5] McDonagh E.M. , Trynka G., McCarthy M., Holzinger E.R., Khader S., Nakic N., Hu X., Cornu H., Dunham I., Hulcoop D. Human Genetics and Genomics for Drug Target Identification and Prioritization: Open Targets’ Perspective. Annu Rev Biomed Data Sci. 2024; 7:59–81. 38608311 10.1146/annurev-biodatasci-102523-103838

[6] Piñero J. , Ramírez-Anguita J.M., Saüch-Pitarch J., Ronzano F., Centeno E., Sanz F., Furlong L.I. The DisGeNET knowledge platform for disease genomics: 2019 update. Nucleic Acids Res. 2020; 48:D845–D855. 31680165 10.1093/nar/gkz1021 PMC7145631

[7] Fang H. , Knight J.C. Priority index: database of genetic targets in immune-mediated disease. Nucleic Acids Res. 2022; 50:D1358–D1367. 34751399 10.1093/nar/gkab994 PMC8728240

[8] di Micco P. , Antolin A.A., Mitsopoulos C., Villasclaras-Fernandez E., Sanfelice D., Dolciami D., Ramagiri P., Mica I.L., Tym J.E., Gingrich P.W. et al. canSAR: update to the cancer translational research and drug discovery knowledgebase. Nucleic Acids Res. 2023; 51:D1212–D1219. 36624665 10.1093/nar/gkac1004 PMC9825411

[9] De Cesco S. , Davis J.B., Brennan P.E. TargetDB: A target information aggregation tool and tractability predictor. PLoS One. 2020; 15:e0232644. 32877430 10.1371/journal.pone.0232644 PMC7467329

[10] Ochoa D. , Hercules A., Carmona M., Suveges D., Baker J., Malangone C., Lopez I., Miranda A., Cruz-Castillo C., Fumis L. et al. The next-generation Open Targets Platform: reimagined, redesigned, rebuilt. Nucleic Acids Res. 2023; 51:D1353–D1359. 36399499 10.1093/nar/gkac1046 PMC9825572

[11] Koscielny G. , An P., Carvalho-Silva D., Cham J.A., Fumis L., Gasparyan R., Hasan S., Karamanis N., Maguire M., Papa E. et al. Open Targets: a platform for therapeutic target identification and validation. Nucleic Acids Res. 2017; 45:D985–D994. 27899665 10.1093/nar/gkw1055 PMC5210543

[12] Harrison P.W. , Amode M.R., Austine-Orimoloye O., Azov A.G., Barba M., Barnes I., Becker A., Bennett R., Berry A., Bhai J. et al. Ensembl 2024. Nucleic Acids Res. 2024; 52:D891–D899. 37953337 10.1093/nar/gkad1049 PMC10767893

[13] Malone J. , Holloway E., Adamusiak T., Kapushesky M., Zheng J., Kolesnikov N., Zhukova A., Brazma A., Parkinson H. Modeling sample variables with an Experimental Factor Ontology. Bioinformatics. 2010; 26:1112–1118. 20200009 10.1093/bioinformatics/btq099 PMC2853691

[14] Carvalho-Silva D. , Pierleoni A., Pignatelli M., Ong C., Fumis L., Karamanis N., Carmona M., Faulconbridge A., Hercules A., McAuley E. et al. Open Targets Platform: new developments and updates two years on. Nucleic Acids Res. 2019; 47:D1056–D1065. 30462303 10.1093/nar/gky1133 PMC6324073

[15] Trajanoska K. , Bhérer C., Taliun D., Zhou S., Richards J.B., Mooser V. From target discovery to clinical drug development with human genetics. Nature. 2023; 620:737–745. 37612393 10.1038/s41586-023-06388-8

[16] Ochoa D. , Karim M., Ghoussaini M., Hulcoop D.G., McDonagh E.M., Dunham I. Human genetics evidence supports two-thirds of the 2021 FDA-approved drugs. Nat. Rev. Drug Discov. 2022; 21:551. 35804044 10.1038/d41573-022-00120-3

[17] Ghoussaini M. , Mountjoy E., Carmona M., Peat G., Schmidt E.M., Hercules A., Fumis L., Miranda A., Carvalho-Silva D., Buniello A. et al. Open Targets Genetics: systematic identification of trait-associated genes using large-scale genetics and functional genomics. Nucleic Acids Res. 2021; 49:D1311–D1320. 33045747 10.1093/nar/gkaa840 PMC7778936

[18] Kurki M.I. , Karjalainen J., Palta P., Sipilä T.P., Kristiansson K., Donner K.M., Reeve M.P., Laivuori H., Aavikko M., Kaunisto M.A. et al. FinnGen provides genetic insights from a well-phenotyped isolated population. Nature. 2023; 613:508–518. 36653562 10.1038/s41586-022-05473-8 PMC9849126

[19] GTEx Consortium The GTEx Consortium atlas of genetic regulatory effects across human tissues. Science. 2020; 369:1318–1330. 32913098 10.1126/science.aaz1776 PMC7737656

[20] Mountjoy E. , Schmidt E.M., Carmona M., Schwartzentruber J., Peat G., Miranda A., Fumis L., Hayhurst J., Buniello A., Karim M.A. et al. An open approach to systematically prioritize causal variants and genes at all published human GWAS trait-associated loci. Nat. Genet. 2021; 53:1527–1533. 34711957 10.1038/s41588-021-00945-5 PMC7611956

[21] Wang Q. , Dhindsa R.S., Carss K., Harper A.R., Nag A., Tachmazidou I., Vitsios D., Deevi S.V.V., Mackay A., Muthas D. et al. Rare variant contribution to human disease in 281,104 UK Biobank exomes. Nature. 2021; 597:527–532. 34375979 10.1038/s41586-021-03855-y PMC8458098

[22] Riveros-Mckay F. , Oliver-Williams C., Karthikeyan S., Walter K., Kundu K., Ouwehand W.H., Roberts D., Di Angelantonio E., Soranzo N., Danesh J. et al. The influence of rare variants in circulating metabolic biomarkers. PLoS Genet. 2020; 16:e1008605. 32150548 10.1371/journal.pgen.1008605 PMC7108731

[23] Singh T. , Poterba T., Curtis D., Akil H., Al Eissa M., Barchas J.D., Bass N., Bigdeli T.B., Breen G., Bromet E.J. et al. Rare coding variants in ten genes confer substantial risk for schizophrenia. Nature. 2022; 604:509–516. 35396579 10.1038/s41586-022-04556-w PMC9805802

[24] Makarious M.B. , Lake J., Pitz V., Ye Fu A., Guidubaldi J.L., Solsberg C.W., Bandres-Ciga S., Leonard H.L., Kim J.J., Billingsley K.J. et al. Large-scale rare variant burden testing in Parkinson's disease. Brain. 2023; 146:4622–4632. 37348876 10.1093/brain/awad214 PMC10629770

[25] Soh P.X.Y. , Mmekwa N., Petersen D.C., Gheybi K., van Zyl S., Jiang J., Patrick S.M., Campbell R., Jaratlerdseri W., Mutambirwa S.B.A. et al. Prostate cancer genetic risk and associated aggressive disease in men of African ancestry. Nat. Commun. 2023; 14:8037. 38052806 10.1038/s41467-023-43726-w PMC10697980

[26] Martin A.R. , Williams E., Foulger R.E., Leigh S., Daugherty L.C., Niblock O., Leong I.U.S., Smith K.R., Gerasimenko O., Haraldsdottir E. et al. PanelApp crowdsources expert knowledge to establish consensus diagnostic gene panels. Nat. Genet. 2019; 51:1560–1565. 31676867 10.1038/s41588-019-0528-2

[27] Thormann A. , Halachev M., McLaren W., Moore D.J., Svinti V., Campbell A., Kerr S.M., Tischkowitz M., Hunt S.E., Dunlop M.G. et al. Flexible and scalable diagnostic filtering of genomic variants using G2P with Ensembl VEP. Nat. Commun. 2019; 10:2373. 31147538 10.1038/s41467-019-10016-3 PMC6542828

[28] Shen A. , Barbero M.C., Koylass B., Tsukanov K., Cezard T., Keane T.M. CMAT: ClinVar Mapping and Annotation Toolkit. Bioinform Adv. 2024; 4:vbae018. 38384863 10.1093/bioadv/vbae018 PMC10879749

[29] Landrum M.J. , Chitipiralla S., Brown G.R., Chen C., Gu B., Hart J., Hoffman D., Jang W., Kaur K., Liu C. et al. ClinVar: improvements to accessing data. Nucleic Acids Res. 2020; 48:D835–D844. 31777943 10.1093/nar/gkz972 PMC6943040

[30] Sondka Z. , Dhir N.B., Carvalho-Silva D., Jupe S. MadhumitaMadhumita McLaren K., Starkey M., Ward S., Wilding J., Ahmed M. et al. COSMIC: a curated database of somatic variants and clinical data for cancer. Nucleic Acids Res. 2024; 52:D1210–D1217. 38183204 10.1093/nar/gkad986 PMC10767972

[31] Martínez-Jiménez F. , Muiños F., Sentís I., Deu-Pons J., Reyes-Salazar I., Arnedo-Pac C., Mularoni L., Pich O., Bonet J., Kranas H. et al. A compendium of mutational cancer driver genes. Nat. Rev. Cancer. 2020; 20:555–572. 32778778 10.1038/s41568-020-0290-x

[32] Pacini C. , Duncan E., Gonçalves E., Gilbert J., Bhosle S., Horswell S., Karakoc E., Lightfoot H., Curry E., Muyas F. et al. A comprehensive clinically informed map of dependencies in cancer cells and framework for target prioritization. Cancer Cell. 2024; 42:301–316. 38215750 10.1016/j.ccell.2023.12.016

[33] Tian R. , Abarientos A., Hong J., Hashemi S.H., Yan R., Dräger N., Leng K., Nalls M.A., Singleton A.B., Xu K. et al. Genome-wide CRISPRi/a screens in human neurons link lysosomal failure to ferroptosis. Nat. Neurosci. 2021; 24:1020–1034. 34031600 10.1038/s41593-021-00862-0 PMC8254803

[34] Groza T. , Gomez F.L., Mashhadi H.H., Muñoz-Fuentes V., Gunes O., Wilson R., Cacheiro P., Frost A., Keskivali-Bond P., Vardal B. et al. The International Mouse Phenotyping Consortium: comprehensive knockout phenotyping underpinning the study of human disease. Nucleic Acids Res. 2023; 51:D1038–D1045. 36305825 10.1093/nar/gkac972 PMC9825559

[35] Stephenson J.D. , Totoo P., Burke D.F., Jänes J., Beltrao P., Martin M.J. ProtVar: mapping and contextualizing human missense variation. Nucleic Acids Res. 2024; 52:W140–W147. 38769064 10.1093/nar/gkae413 PMC11223857

[36] Cheng J. , Novati G., Pan J., Bycroft C., Žemgulytė A., Applebaum T., Pritzel A., Wong L.H., Zielinski M., Sargeant T. et al. Accurate proteome-wide missense variant effect prediction with AlphaMissense. Science. 2023; 381:eadg7492. 37733863 10.1126/science.adg7492

[37] Varadi M. , Anyango S., Deshpande M., Nair S., Natassia C., Yordanova G., Yuan D., Stroe O., Wood G., Laydon A. et al. AlphaFold Protein Structure Database: massively expanding the structural coverage of protein-sequence space with high-accuracy models. Nucleic Acids Res. 2022; 50:D439–D444. 34791371 10.1093/nar/gkab1061 PMC8728224

[38] Schymkowitz J. , Borg J., Stricher F., Nys R., Rousseau F., Serrano L. The FoldX web server: an online force field. Nucleic Acids Res. 2005; 33:W382–8. 15980494 10.1093/nar/gki387 PMC1160148

[39] Ferguson C. , Araújo D., Faulk L., Gou Y., Hamelers A., Huang Z., Ide-Smith M., Levchenko M., Marinos N., Nambiar R. et al. Europe PMC in 2020. Nucleic Acids Res. 2021; 49:D1507–D1514. 33180112 10.1093/nar/gkaa994 PMC7778976

[40] Vaswani A. , Shazeer N., Parmar N., Uszkoreit J., Jones L., Gomez A.N., Kaiser Ł., Polosukhin I. Attention is all you need. Proceedings of the 31st International Conference on Neural Information Processing Systems, NIPS’17. 2017; Red Hook, NY, USA Curran Associates Inc 6000–6010.

[41] Mendez D. , Gaulton A., Bento A.P., Chambers J., De Veij M., Félix E., Magariños M.P., Mosquera J.F., Mutowo P., Nowotka M. et al. ChEMBL: towards direct deposition of bioassay data. Nucleic Acids Res. 2019; 47:D930–D940. 30398643 10.1093/nar/gky1075 PMC6323927

[42] Sollis E. , Mosaku A., Abid A., Buniello A., Cerezo M., Gil L., Groza T., Güneş O., Hall P., Hayhurst J. et al. The NHGRI-EBI GWAS Catalog: knowledgebase and deposition resource. Nucleic Acids Res. 2023; 51:D977–D985. 36350656 10.1093/nar/gkac1010 PMC9825413

[43] UniProt Consortium The , Bateman A., Martin M.-J., Orchard S., Magrane M., Ahmad S., Alpi E., Bowler-Barnett E.H., Britto R., Bye-A-Jee H. et al. UniProt: the Universal Protein Knowledgebase in 2023. Nucleic Acids Res. 2022; 51:D523–D531. 10.1093/nar/gkac1052 PMC9825514 36408920

[44] Karczewski K.J. , Francioli L.C., Tiao G., Cummings B.B., Alföldi J., Wang Q., Collins R.L., Laricchia K.M., Ganna A., Birnbaum D.P. et al. The mutational constraint spectrum quantified from variation in 141,456 humans. Nature. 2020; 581:434–443. 32461654 10.1038/s41586-020-2308-7 PMC7334197

[45] George N. , Fexova S., Fuentes A.M., Madrigal P., Bi Y., Iqbal H., Kumbham U., Nolte N.F., Zhao L., Thanki A.S. et al. Expression Atlas update: insights from sequencing data at both bulk and single cell level. Nucleic Acids Res. 2024; 52:D107–D114. 37992296 10.1093/nar/gkad1021 PMC10767917

[46] Sjöstedt E. , Zhong W., Fagerberg L., Karlsson M., Mitsios N., Adori C., Oksvold P., Edfors F., Limiszewska A., Hikmet F. et al. An atlas of the protein-coding genes in the human, pig, and mouse brain. Science. 2020; 367:eaay5947. 32139519 10.1126/science.aay5947

[47] Hunter F.M.I. , Bento A.P., Bosc N., Gaulton A., Hersey A., Leach A.R. Drug Safety Data Curation and Modeling in ChEMBL: Boxed Warnings and Withdrawn Drugs. Chem. Res. Toxicol. 2021; 34:385–395. 33507738 10.1021/acs.chemrestox.0c00296 PMC7888266

[48] Skuta C. , Popr M., Muller T., Jindrich J., Kahle M., Sedlak D., Svozil D., Bartunek P. Probes &Drugs portal: an interactive, open data resource for chemical biology. Nat. Methods. 2017; 14:759–760. 28753599 10.1038/nmeth.4365

[49] Whirl-Carrillo M. , Huddart R., Gong L., Sangkuhl K., Thorn C.F., Whaley R., Klein T.E. An Evidence-Based Framework for Evaluating Pharmacogenomics Knowledge for Personalized Medicine. Clin. Pharmacol. Ther. 2021; 110:563–572. 34216021 10.1002/cpt.2350 PMC8457105

[50] Brennan R.J. , Jenkinson S., Brown A., Delaunois A., Dumotier B., Pannirselvam M., Rao M., Ribeiro L.R., Schmidt F., Sibony A. et al. The state of the art in secondary pharmacology and its impact on the safety of new medicines. Nat. Rev. Drug Discov. 2024; 23:525–545. 38773351 10.1038/s41573-024-00942-3

[51] Baldarelli R.M. , Smith C.L., Ringwald M., Richardson J.E., Bult C.J., Mouse Genome Informatics Group Mouse Genome Informatics: an integrated knowledgebase system for the laboratory mouse. Genetics. 2024; 227:iyae031. 38531069 10.1093/genetics/iyae031 PMC11075557

[52] Wilkinson M.D. , Dumontier M., Aalbersberg I.J.J., Appleton G., Axton M., Baak A., Blomberg N., Boiten J.-W., da Silva Santos L.B., Bourne P.E. et al. The FAIR Guiding Principles for scientific data management and stewardship. Sci Data. 2016; 3:160018. 26978244 10.1038/sdata.2016.18 PMC4792175

[53] Margoni T. , Peters D. Creative Commons Licenses: Empowering Open Access. 2016; 10.2139/ssrn.2746044.

[54] Feizi A. , Ray K. otargen: GraphQL-based R package for tidy data accessing and processing from Open Targets Genetics. Bioinformatics. 2023; 39:btad441. 37467069 10.1093/bioinformatics/btad441 PMC10394122

[55] Dwane L. , Behan F.M., Gonçalves E., Lightfoot H., Yang W., van der Meer D., Shepherd R., Pignatelli M., Iorio F., Garnett M.J. Project Score database: a resource for investigating cancer cell dependencies and prioritizing therapeutic targets. Nucleic Acids Res. 2020; 49:D1365–D1372. 10.1093/nar/gkaa882 PMC7778984 33068406

[56] Rusina P.V. , Falaguera M.J., Romero J.M.R., McDonagh E.M., Dunham I., Ochoa D. Genetic support for FDA-approved drugs over the past decade. Nat. Rev. Drug Discov. 2023; 22:864. 37803084 10.1038/d41573-023-00158-x

[57] Zhou Y. , Zhang Y., Zhao D., Yu X., Shen X., Zhou Y., Wang S., Qiu Y., Chen Y., Zhu F. TTD: Therapeutic Target Database describing target druggability information. Nucleic Acids Res. 2024; 52:D1465–D1477. 37713619 10.1093/nar/gkad751 PMC10767903

[58] Raies A. , Tulodziecka E., Stainer J., Middleton L., Dhindsa R.S., Hill P., Engkvist O., Harper A.R., Petrovski S., Vitsios D. DrugnomeAI is an ensemble machine-learning framework for predicting druggability of candidate drug targets. Communications Biology. 2022; 5:1–16. 36434048 10.1038/s42003-022-04245-4 PMC9700683

[59] Hasselgren C. , Oprea T.I. Artificial Intelligence for Drug Discovery: Are We There Yet. Annu. Rev. Pharmacol. Toxicol. 2024; 64:527–550. 37738505 10.1146/annurev-pharmtox-040323-040828

[60] Bjornsdottir G. , Chalmer M.A., Stefansdottir L., Skuladottir A.T., Einarsson G., Andresdottir M., Beyter D., Ferkingstad E., Gretarsdottir S., Halldorsson B.V. et al. Rare variants with large effects provide functional insights into the pathology of migraine subtypes, with and without aura. Nat. Genet. 2023; 55:1843–1853. 37884687 10.1038/s41588-023-01538-0 PMC10632135

[61] Zhou H. , Kember R.L., Deak J., Xu H., Toikumo S., Yuan K., Lind P.A., Farajzadeh L., Wang L., Hatoum A.S. et al. Multi-ancestry study of the genetics of problematic alcohol use in over 1 million individuals. Nat. Med. 2023; 29:3184–3192. 38062264 10.1038/s41591-023-02653-5 PMC10719093

