# Supplementary material for: Open Targets Platform: facilitating therapeutic hypotheses building in drug discovery
Source: Nucleic Acids Res. 2024 Dec 6;53(D1):D1467–75. doi: 10.1093/nar/gkae1128 (PMC11701534; doi:10.1093/nar/gkae1128)
Supplement: gkae1128_Supplemental_File [file gkae1128_supplemental_file.docx]

| **Total number of** | **22.09** | **24.09** |
| --- | --- | --- |
| Evidence strings | 14,229,684 | 17,853,184 |
| Target-disease associations | 7,003,171 | 8,155,988 |
| Targets | 61,888 | 63,121 |
| Diseases | 20,931 | 28,327 |
| Drugs | 12,854 | 18,041 |

Supplementary table 1: Quantitative view of the Platform data expansion since the last NAR report (comparison between 22.09 and 24.09 releases).
